# Evaluating Cytotoxicity of Hyaluronate Targeted Solid Lipid Nanoparticles of Etoposide on SK-OV-3 Cells

**DOI:** 10.1155/2014/746325

**Published:** 2014-04-24

**Authors:** Parviz Mohammadi Ghalaei, Jaleh Varshosaz, Hojatollah Sadeghi Aliabadi

**Affiliations:** ^1^Department of Pharmaceutics, School of Pharmacy and Novel Drug Delivery Systems Research Centre, Isfahan University of Medical Sciences, P.O. Box 81745-359, Isfahan 81746-73461, Iran; ^2^Department of Pharmaceutical Biotechnology, School of Pharmacy and Pharmaceutical Sciences, Isfahan University of Medical Sciences, Isfahan 81746-73461, Iran

## Abstract

The epithelial ovarian carcinoma is one of the most fatal gynecological cancers. Etoposide is used in treating platinum-resistant ovarian cancer. Sodium hyaluronate is a substance that binds to the CD_44_ receptors overexpressed in SK-OV-3 cells of epithelial ovarian carcinoma. The aim of the present work was to study the cytotoxicity effect of hyaluronate targeted solid lipid nanoparticles (SLNs) of etoposide on SK-OV-3 cells. The cytotoxicity of the targeted and nontargeted SLNs of etoposide was compared to free drug on the SK-OV-3 cells by MTT assay method. The cellular uptake of the targeted and nontargeted nanoparticles containing sodium fluorescein was also studied. The difference of cell vitality between nontargeted nanoparticles and also targeted nanoparticles with free drug was significant. Targeted nanoparticles also caused more toxicity than nontargeted nanoparticles (*P* < 0.05). After 4 hours of incubating, the fluorescence was remarkably higher in the cells treated by targeted SLNs rather than nontargeted ones, and there was no observable fluorescence in cells incubated with pure sodium fluorescein. Hyaluronate targeted SLNs containing etoposide increased the cytotoxicity of etoposide on SK-OV-3 cells which may be a worthwhile potential method for reducing the prescribed dose and systemic side effects of this drug in epithelial ovarian carcinoma.

## 1. Introduction

The epithelial ovarian carcinoma is one of the most fatal gynecological cancers across the globe. In spite of early recovery by surgical and chemotherapy treatments, the 5-year survival rate for the patients is only 13 percent. The database GLOBCAN related to the World Health Organization (WHO) has reported incidence of about 192000 cases in the world, in the year of 2000. 6000 cases of the mentioned cases have occurred in the UK, and 21000 cases in the U.S. For treating the disease, the tumor will be removed by surgical procedures and then chemotherapy would be started with platinum-based chemotherapy (cisplatin and carboplatin), which treating regime includes cisplatin and carboplatin with the drugs such as paclitaxel, docetaxel, cyclophosphamide, and doxorubicin. In some of the patients, the disease relapses after 6 months of chemotherapy; this condition is defined as platinum resistant, in which treatment would be continued with drugs such as topotecan and etoposide [1].

Etoposide, as other chemotherapy agents, has many side effects such as bone marrow suppression, granulocytopenia, thrombocytopenia [2], mucositis, moderate to severe esophagitis, hepatotoxicity, metabolic acidosis, and anemia [3].

The complications of anticancer drugs have caused scientists to try two approaches to solve the problem: developing new drugs with fewer side effects and application of new drug delivery systems with high specificity to cancerous tissues; the second approach has lower costs and more attention nowadays. Solid lipid nanoparticles (SLNs) are one of the most important nanosized drug delivery systems that were introduced about two decades ago [4].

SLNs that are often considered for intravenous application are colloidal submicron carriers sized 50 to 1000 nm and composed of solid lipids dispersed in water or surfactant aqueous solution. These nanoparticles have particular features like small size, high surface area, and high loading of drug that makes them potent and beneficial carriers for improving drug efficacy [5, 6]. SLNs are similar to o/w emulsions used for total parenteral nutrition; the difference is that emulsion liquid lipid has been replaced with a solid lipid. SLNs have advantages such as controlled drug release in considered site, excellent biocompatibility, increase in drug stability, high drug content, easy industrialization and sterilization, better control of drug release kinetics, high bioavailability for bioactive drugs, chemical protection of sensitive drugs, easier producing rather than biopolymeric nanoparticles, producible by common emulsification methods, long-time stability, and various applications [4, 7, 8].

For parenteral administration, SLN dispersions must be sterile. SLNs with appropriately small particle size less than 200 nm can be sterilized using filtration. Autoclaving the finished dispersion is not practical as the lipids melt at sterilizing temperatures and the molten lipid droplets coalesce. Therefore just aseptic manufacturing processes following sterilization of the starting materials by gamma irradiation of the final dispersion or exposure to ethylene oxide (EO) gas are applicable for their sterilization. Bacterial endotoxins in raw materials need to be monitored, especially when raw materials are of natural origin. It may be possible to lyophilize the SLN dispersions, and this lyophile can be irradiated or exposed to EO.

SLNs are used in transdermal applications, as gene vector carriers, for topical uses, as cosmeceuticals, as targeted carriers of anticancer drugs to solid tumors, in breast cancer and lymph node metastases and in antitubercular chemotherapy.

So far successful studies have been performed upon nanoparticles containing etoposide. For example, the study of Yadav et al. [9] was performed in the survey of poly(lactic-coglycolic acid)-monomethoxy-poly(polyethylene glycol) and poly(lactic-coglycolic acid)-Pluronic block copolymers and the study of Reddy et al. [10] on nanoparticles produced by tripalmitin could be mentioned.

**Figure 1:**
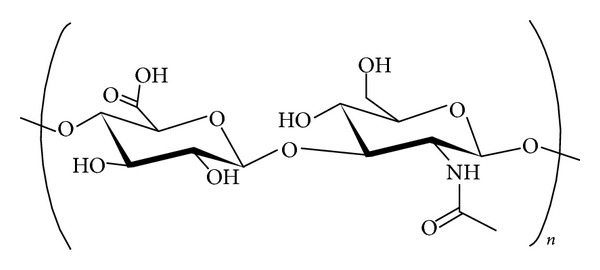
Chemical structure of hyaluronan: polymeric repeat of* D*-glucuronic acid and* N*-acetylglucosamine.

Hyaluronan (Figure 1), available in the market as sodium hyaluronate (HA), is a high molecular weight glycosaminoglycan present in extracellular matrix and is necessary for cellular growth and structural stability of organs and tissue structure.

HA regulates cell proliferation and movements by interacting with CD_44_ receptors and receptor for HA mediated motility (RHAMM). Because of overexpression of CD_44_ receptors by cancer cells, interfering in CD_44_-HA interaction by targeting drugs at CD_44_ is an effective strategy to treat cancers. HA bound to nanoparticles, in addition to its targeting role, may act as a protecting agent of nanoparticles against body phagocytosis system [11–13]. The mentioned method has been used to deliver agents such as doxorubicin [14], epirubicin [15], paclitaxel [16], mitomycin C [17], SiRNA [18], and DNA [19].

To our knowledge there is not any report on the application of the hyaluronate targeted SLNs in drug delivery of etoposide in SK-OV-3 cells although there are some studies on the hyaluronate targeted SLNs. This study alongside with thousands of similar ones could help to introduce new clinically applicable drug delivery systems with appropriate physicochemical properties, successful targeting, and enhanced cytotoxicity in the future. This study was performed in order to evaluate cytotoxicity of HA targeted SLNs containing etoposide, prepared and optimized in our previous study [20] in SK-OV-3 cells.

## 2. Materials and Methods

### 2.1. Materials

Stearylamine (SA), dodecylamine (DDA), cetyl alcohol, dialysis bags with molecular weight cut-off of 12400 Da, and thiazolyl blue tetrazolium bromide (MTT) were from Sigma-Aldrich Company (US). Acetone, dichloromethane, and Tween 80 were from Merck Chemical Company (Germany). RPMI 1640 culture medium, penicillin-streptomycin, and fetal bovine serum were from PAA Company, Austria. Etoposide was a gift from Nippon Kayaku Co, Ltd. (Tokyo, Japan). Sodium hyaluronate (Mw = 6,400 Da) was from Lifecore Biomedical (US) and SK-OV-3 cells were from Pasteur Institute (Iran).

### 2.2. Preparing Nanoparticles

SLNs were produced by emulsification-solvent evaporation method. According to the results of our previous study [20], the lipid phase including 30 mg etoposide, 30 mg cetyl alcohol, and 30 mg SA was dissolved in 1.8 mL of 1 : 1 mixture of acetone-dichloromethane. Then the mentioned solution was added during 3 minutes to the 18 mL of Tween 80 solution (1% w/v) in deionized water, while stirring in 1200 rpm. Ultimately, produced nanoemulsion was stirred in 600 rpm in room temperature for 75 minutes to evaporate the solution [21]. The blank nanoparticles were produced by the same method but without etoposide.

### 2.3. Physical Binding of HA to the SLNs Surface

After 15 minutes of adding organic phase to aqueous phase, HA dissolved in deionized water containing Tween 80 (1% w/v) was added to nanoparticles mixture during 5 minutes, while stirring at 600 rpm, in order to produce targeted nanoparticles [22].

Nonbound HA was separated from nanoparticles mixture by dialyzing versus 100 mL deionized water containing Tween 80 (1% w/v) using dialysis bag with molecular weight cut-off of 12,400 Da for 40 minutes so that the deionized water containing Tween 80 (1% w/v) was replaced every 10 minutes. To determine the amount of HA bounded to SLNs after separation of unbound HA, some part of the targeted nanoparticles mixture was dried under vacuum and subjected to elemental analysis (CHN) (CHNS-932, Leco, USA) and, by subtracting the total amount of HA from gaining value, the amount of HA bound on the SLNs surface was calculated.

### 2.4. Measuring Particle Size, Polydispersity Index, and Zeta Potential

The particle size, polydispersity index, and zeta potential of nanoparticles were measured by a Zetasizer (Zetasizer 3000; Malvern Instruments, Malvern, UK), after 1 : 10 diluting the samples with deionized water.

### 2.5. Determining Drug Loading and Release

The loading efficiency percent was determined by centrifugation (Eppendorf 5430 centrifuge, Germany). The dispersion of nanoparticles was poured in centrifugal filter tubes (Amicon Ultra, Ireland) with a 10 kDa molecular weight cutoff to separate the aqueous medium [23]. The concentration of free etoposide in the filtrate was determined by measuring its absorption in 276.4 nm (UV-VIS spectrophotometer, Shimadzu Scientific Instruments, Japan) and converting the absorbance to concentration using the calibration equation of etoposide in aqueous phase containing 1% w/v of Tween 80. The amount of encapsulated drug was computed indirectly by calculating the difference between the total amounts of drug used in preparation of nanoparticles and the free drug. Ultimately, loading efficiency percent was computed by the following equation:

(1) Loding  efficiency  percent =(total  drug  weight−free  drug  weight)total  drug  weight×100.

Drug release profiles from the NPLs were determined in phosphate buffer saline (PBS, 0.01 M, pH 7.4 containing 1% w/v Tween 80) at 37°C. A total of 2 mL of NPLs suspension was placed in dialysis bag with molecular weight cut-off of 12,400 Da and suspended in a beaker containing 50 mL of PBS on a magnetic stirrer with a speed of 200 rpm. Samples were withdrawn periodically and replaced with the same volume of PBS at the same temperature. The content of etoposide in the samples was determined spectrophotometrically at 268.7 nm.

### 2.6. MTT Colorimetric Cytotoxicity Assay

To determine cell proliferation, an MTT assay was carried out. A total of 180 *μ*L of the cell suspension (5 × 10^4^ cells/mL) were placed in each well of a 96-well plate except for one row for blank that was filled by an equal amount of medium. After a 24 h period of incubation at 37°C in a CO_2_ incubator with 5% CO_2_ and 95% humidity, all 4 wells of cells were treated with 20 *μ*L of one of the concentrations of etoposide as much as 0.475, 0.95, 1.9, and 3.8 *μ*M of etoposide. The IC_50_ of etoposide for SK-OV-3 cells was determined to be 1.9 *μ*M [24]. In order to assure that microorganisms would not be able to contaminate the SLNs and interfere with cytotoxicity results, preparation of solution of free drug and also preparation and dilution of SLNs suspensions were carried out in aseptic conditions under a laminar flow hood. It should be pointed out that solutions of organic and aqueous phases were presterilized by ultraviolet germicidal irradiation method.

Treated groups included either a solution of free drug in 1 w/v% aqueous solution of Tween 80 or encapsulated drug in nontargeted and targeted nanoparticles, with blanks of nontargeted and targeted nanoparticles, while culture medium and Tween 80 1 w/v% (each one in 8 wells) serve as control groups. The cells were incubated for further 48 h. After the treatment, 20 *μ*L/well of the MTT solution (5 mg/mL of PBS) was added to the cells and incubated for 3 h; then the supernatant was removed carefully and the formazan crystals were dissolved by adding 150 *μ*L of DMSO. Finally, the absorbance of each well was measured at 570 nm by an ELIZA plate reader (STAT FAX 2100 Microplate Reader, Awareness Technology, Inc., US). The effect of each treatment on cell viability was calculated by comparing the relative absorbance of treated cells against the respective controls, using the following equation [25]:

(2) Cell  survival  % =(mean  absorbance  of  each  group    − mean  absorbance  of  blank)  ×(mean  absorbance  of  negative  control     − mean  absorbance  of  blank)−1  ×100.

### 2.7. Qualitative Comparison of Drug Uptake from Nanoparticles by Fluorescence Imaging

First, 2700 *μ*L of the cellular suspension with the concentration of  10^5^ cells/mL was poured into 10 wells of a 12-well plate containing lamels at the bottom and then incubated for 48 h in CO_2_ incubator. Then the nontargeted and targeted nanoparticles were loaded with sodium fluorescein instead of etoposide by the same method as mentioned above for drug-loaded SLNs. The final concentration of loaded sodium fluorescein in nanoparticles was 1 mg/mL. Blank nanoparticles were also prepared but without sodium fluorescein. To prepare free sodium fluorescein solution, 10 *μ*L of stock solution (100 mg/mL) was diluted to 1 mL to provide the final concentration of 1 mg/mL.

Finally, 300 *μ*L of each sample was added to 2 wells (one for imaging in the 1st hour and the other for imaging in 4th hour) and was incubated. Lamels were withdrawn and imaging was performed by visible fluorescence microscope (Olympus, IX71, Japan) [11].

### 2.8. Statistical Analysis

All data are the results of three separate experiments, and the results are expressed as the mean ± standard deviation (*n* = 3). Statistical analysis was performed using one-way analysis of variance (ANOVA) and an independent Student's *t*-test with the SPSS software (version 18, US). A *P* value of less than 0.05 was considered significant.

## 3. Results and Discussion

### 3.1. Physicochemical Properties of Nanoparticles

**Figure 2:**
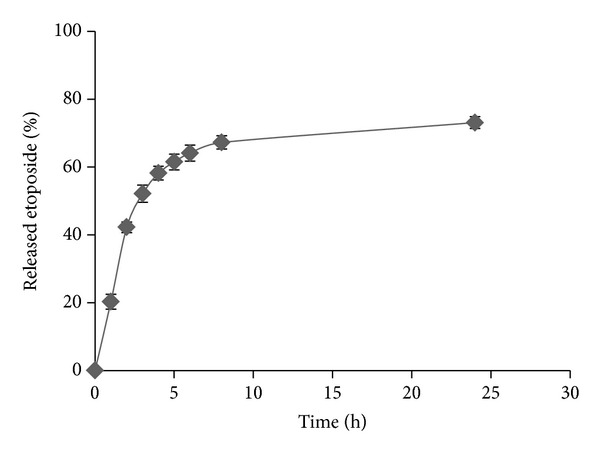
Etoposide release profile from HA targeted SLNs.

Table 1 represents properties of nanoparticles. The particle size of nontargeted and targeted SLNs was 179.6 ± 16.31 and 416.42 ± 31.85, respectively. Zeta potential of nontargeted SLNs was 11.82 ± 0.52 that changed to −12.65 ± 0.49 after coating with HA. Drug loading efficiency was about 64.92 ± 3.76% and release efficiency percent in 24 h was 65.47 ± 4.68% which is an acceptable value. HA was coated as much as 55.89 percent on the SLNs.  Figure 2 represents drug release profile from HA targeted nanoparticles.

SLNs have generally long-term stability (about 1–3 years) as small particle size and density close to unity of SLNs mean that the gravity has little effect on particles in dispersion and the Brownian motion is sufficient to maintain colloidal dispersions without creaming or sedimentation. In the present study the presence of physically bound HA and the negative zeta potential of targeted SLNs may seem to threaten stability, but our unpublished results showed that properties of the mentioned SLNs suspension did not change significantly within 10 days. However, as freeze-drying is a suitable method to prevent the Ostwald ripening and avoid aggregation of SLNs, we also dried the nontargeted and targeted SLNs under vacuum with 5% glycerol serving as cryoprotectant and then recovered them by adding deionized water. The results showed that nontargeted SLNs only needed 5 minutes of stirring at 800 rpm and targeted SLNs needed twice the stirring at 800 rpm each time for 3 minutes and then 10 seconds of sonication at a power of 30 w, to retrieve their primary properties. Nonetheless, the SLNs which were used for cytotoxicity study were prepared fresh.

The observed release rate (64.1% in the first 6 hours and 73.1% in 24 hours) could provide appropriate serum concentrations for routine chemotherapy schedules in which the drug (with an* iv* half-life of 6–12 hours) is administered once daily. Also the mean diameter of typically 200–400 nm is well below the size of the smallest blood capillaries in the range of 5-6 *μ*m. Furthermore, because of the heterogeneity of tumors and dynamic status of each tumor, it will be very difficult to assume any maximum single value for particles to exploit the enhanced permeation and retention (EPR) effect. However, the study of Bae and Park suggests that the porosity of the blood vessels in tumors is around 400 nm [26]. A tumor-dependent functional pore cutoff size ranges from 200 nm to 1.2 *μ*m, but the pore cutoff size of porous blood vessels in the majority of tumors is known to be 380–780 nm [27]. Thus, the range of the EPR effect should be similar. Sterically stabilized liposomes of 400 nm in diameter were able to penetrate into tumor interstitium [28]. Accumulation of hyaluronic acid-coated self-assembled nanoparticles with particle size of 400 nm has been reported in the tumor tissue too [29].

### 3.2. Cell Proliferation Assay (MTT Assay)

**Figure 3:**
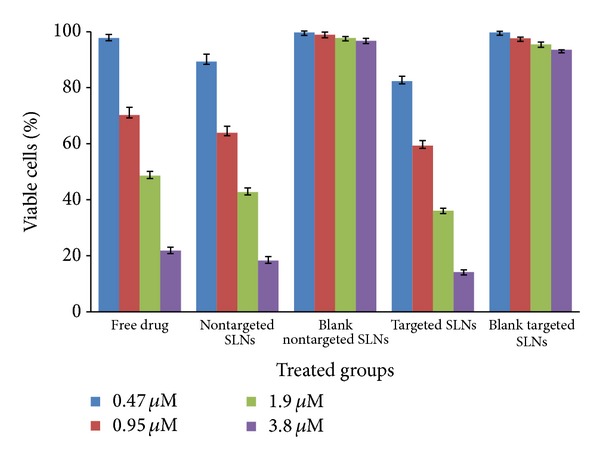
Percentage of viable cells of SK-OV-3 determined by the MTT assay after treatment with etoposide loaded nontargeted and hyaluronate targeted SLNs in comparison to blank nontargeted and targeted SLNs and free drug (*n* = 3).

The obtained results of MTT cytotoxicity assay have been illustrated in Table 2 and Figure 3.

All drug-loaded nanoparticles caused higher cytotoxicity compared to the free etoposide at the same concentration and their respective blank SLNs. The mechanism of enhanced cytotoxicity of drug-loaded lipid nanoparticles has been previously reported [30, 31]. It is well understood that improvement in the cytotoxicity is because of the elevated drug concentrations within the cells. As we can see in Figure 3, nontargeted drug-loaded SLNs have lower cell survival compared to the free etoposide solution. For example, the observed cell survival after treatment with targeted nanoparticles was 36.08 ± 0.88%, while it was 42.73 ± 1.49% and 48.57 ± 1.61% for nontargeted SLNs and free drug solution, respectively, at the concentration of 1.9 *μ*M (*P* < 0.05).

The results verified that targeted and nontargeted SLNs of etoposide have reduced IC_50_ to 52% and 83% of free drug, respectively (Table 2). In a study, Saliou et al. [32] reported that lipid nanocapsules of etoposide reduced the IC_50_ of the drug from 100 to 2.5 *μ*M in H209 cells. These lipid nanocapsules also could reduce the IC_50_ of etoposide to about 4–30 times in glioma cell lines [33]. In an experiment conducted by Nasti et al. [34] chitosan/triphosphate nanoparticles coated with HA showed the IC_50_ of about half of the noncoated nanoparticles on murine fibroblasts of L929 and macrophage cells of J774.2. Han et al. [35] successfully overcame on drug resistance of MCF-7/ADR cells with 4.3-fold reduction in IC_50_ of doxorubicin by SiRNA polyamidoamine-hyaluronic acid complex.

It could be concluded that the internalization of the drug into cells was enhanced when the drug was encapsulated in SLNs. This phenomenon might be the result of the high affinity of lipid materials of SLNs for the cell membrane and the nanoscaled size of SLNs.

The correlation between nanoparticles size and intracellular concentration has been observed in the study performed by Zhang et al. [36] and their results indicated that the less the particle size is, the more the intracellular drug concentration and cytotoxicity is.

In addition, comparing the targeted and nontargeted nanoparticles determines that the cytotoxicity in the targeted nanoparticles has been increased, probably due to the presence of HA on targeted nanoparticles which could interact with CD_44_ receptors and make them internalized into cells more easily. Cho et al. [37] have surveyed NPLs containing docetaxel targeted by HA upon cancer cell line MCF-7 and showed that they were endocytosed by CD_44_ receptors.

### 3.3. Cellular Uptake Studies

**Figure 4:**
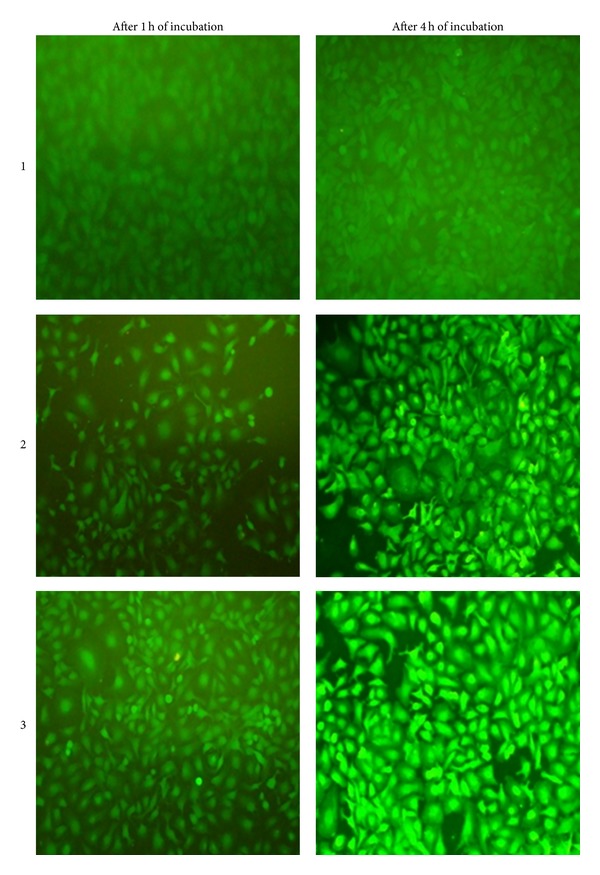
Fluorescence images of SK-OV-3 cells after 1 and 4 hours of incubation with (1) free sodium fluorescein, (2) sodium fluorescein containing nontargeted SLNs, and (3) sodium fluorescein containing targeted SLNs.

After incubating for 1 hour, only targeted nanoparticles made a slight fluorescence in the cells (Figure 4). After 4 hours of incubation, the fluorescence was remarkably higher in the cells which were treated by targeted SLNs rather than those which were treated by nontargeted nanoparticles, and there was no observable fluorescence in cells incubated with pure sodium fluorescein (Figure 4). Therefore, it could be concluded that increased cytotoxicity in results obtained from MTT assay has resulted from special uptake of targeted nanoparticles due to presence of HA as targeting agent.

## 4. Conclusion

Hyaluronate targeted SLNs containing etoposide increase the cytotoxicity of etoposide in SK-OV-3 cells and could be a valuable method for reducing the prescribed dose and also systemic side effects.

## Figures and Tables

**Table 1:** Properties of solid lipid nanoparticles of etoposide.

SLNs type	Particle size (nm)	pdI	zeta potential (mV)	Drug loading efficiency (%)	RE in 24 h (%)
Non-targeted SLNs	179.6 ± 16.3	0.17 ± 0.03	11.82 ± 0.52	—	—
HA targeted SLNs	416.4 ± 31.8	0.30 ± 0.05	−12.65 ± 0.49	64.92 ± 3.76	65.47 ± 4.68

**Table 2:** IC_50 _of etoposide loaded in non-targeted and hyaluronate targeted SLNs in SK-OV-3 cells.

	Free drug	Non-targeted SLNs	AH-targeted SLNs
IC_50_ (*µ*M)	1.49 ± 0.08	1.24 ± 0.13	0.78 ± 0.12
